# Eosinophilic esophageal myositis mimicking achalasia: a rare case of dysphagia with extreme LES pressures and histologic clarity

**DOI:** 10.1007/s12328-025-02209-9

**Published:** 2025-08-30

**Authors:** Matthias Hess, Aart Mookhoek, Johannes Lenglinger, Benjamin Heimgartner, Yves Borbely

**Affiliations:** 1 Department of Visceral Surgery and Medicine, Inselspital, Bern University Hospital, University of Bern, Bern, Switzerland; 2 Institute of Tissue Medicine and Pathology, University of Bern, Bern, Switzerland; 3 Spital Wallis - Spitalzentrum Oberwallis, Klinik Innere Medizin – Gastroenterologie, Visp, Switzerland

**Keywords:** Dysphagia, Eosinophilic myositis esophagus

## Abstract

Eosinophilic infiltration of the esophageal muscular layer, known as eosinophilic esophageal myositis (EoEM), is an exceptionally rare condition that can mimic primary motility disorders such as achalasia. We present the case of a 72-year-old male with progressive dysphagia and significant weight loss, whose high-resolution manometry revealed findings consistent with achalasia, but with unusually elevated lower esophageal sphincter pressures. Surgical myotomy was performed and histopathological analysis unexpectedly revealed intense eosinophilic infiltration of the muscularis propria. The patient was treated with systemic corticosteroids, followed by topical budesonide, leading to partial and temporary clinical improvement. This case highlights eosinophilic esophageal myositis as a potential, but underrecognized differential diagnosis in patients with atypical achalasia features and treatment-refractory dysphagia.

## Introduction:

Eosinophilic infiltration of the gastrointestinal tract is most commonly observed in the mucosal layer, particularly in conditions such as eosinophilic esophagitis (EoE) [1], which is now a well-recognized chronic immune-mediated disease. However, in rare instances, eosinophils can infiltrate deeper layers of the gastrointestinal wall, including the muscularis propria, leading to a distinct and much less understood entity known as eosinophilic esophageal myositis (EoEM) [2, 3].

EoEM differs significantly in its histopathological characteristics and clinical behavior from both EoE and eosinophilic gastroenteritis, which may affect various layers of the gastrointestinal tract but usually involve the mucosa [4]. In contrast, EoEM is characterized by isolated eosinophilic infiltration of the muscular layer of the esophagus, lacking evidence of mucosal inflammation on standard endoscopic biopsies. This anatomical distinction suggests that EoEM may represent a separate disease entity rather than a progression of EoE or eosinophilic gastroenteritis [5].

Clinically, EoEM presents a unique diagnostic challenge, as it may mimic primary esophageal motility disorders such as achalasia [1], both symptomatically and manometrically. Standard diagnostic procedures, including endoscopy with mucosal biopsies and high-resolution manometry, may not be sufficient to identify this condition, often delaying diagnosis. Full-thickness or deep muscular biopsies are typically required to establish the diagnosis, which may only be obtained intraoperatively or via advanced techniques such as endoscopic ultrasound-guided fine-needle aspiration (EUS-FNA) [2, 3, 6].

This case illustrates the diagnostic complexity and therapeutic uncertainty associated with EoEM, particularly in patients with atypical presentations of achalasia. It also underscores the need to recognize eosinophilic esophageal myositis as a potentially distinct clinical entity. We will explore this distinction in greater detail in the discussion, along with a review of similar cases in the literature and their treatment approaches.

## Case presentation

A 72-year-old male with a medical history of spinal neuroforaminal stenosis, hypercholesterolemia, benign prostatic hyperplasia, glaucoma, and a history of hemicolectomy for colorectal carcinoma in 2014 presented with progressively worsening dysphagia to both solids and liquids over several weeks. He reported a significant weight loss of 7 kg over 5 weeks, amounting to more than 10% of his body weight, leading to signs of malnutrition. His only medications in the home setting were esomeprazole 40 mg and tamsulosin 0.4 mg per day. The laboratory tests (complete blood count) showed no abnormalities, especially no eosinophilia.

**Fig. 1:**
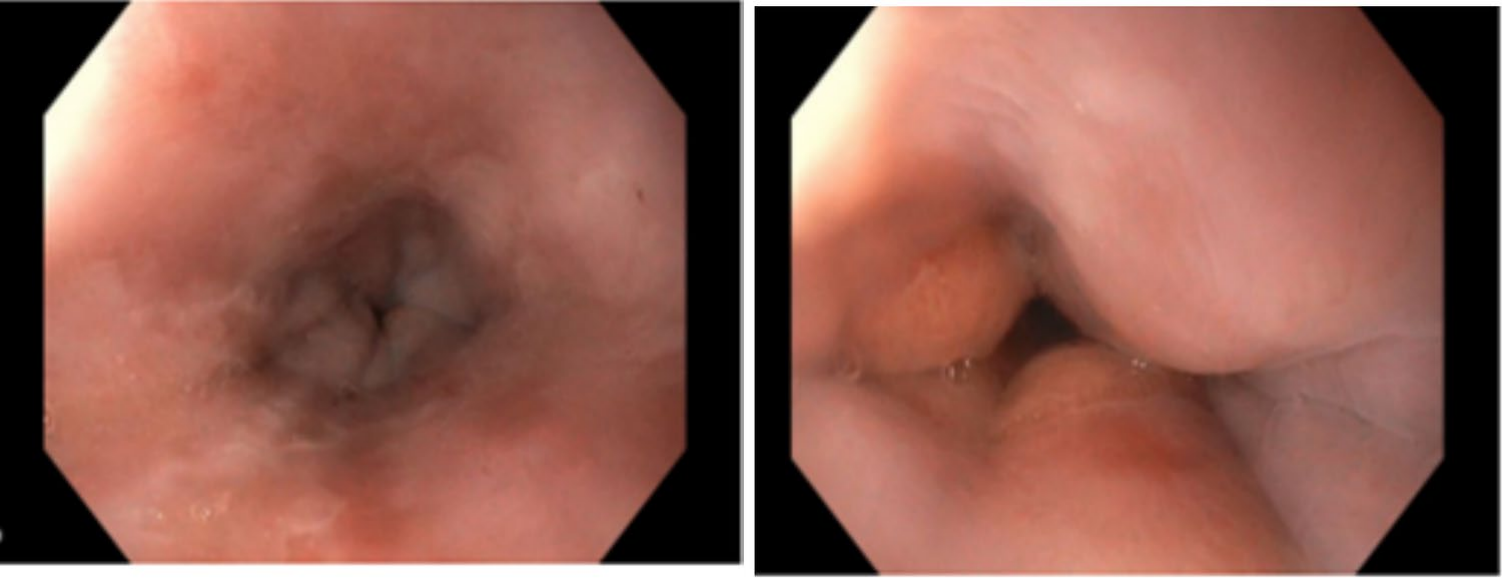
Macroscopic findings on the initial gastroscopy (distal esophagus)

**Fig. 2:**
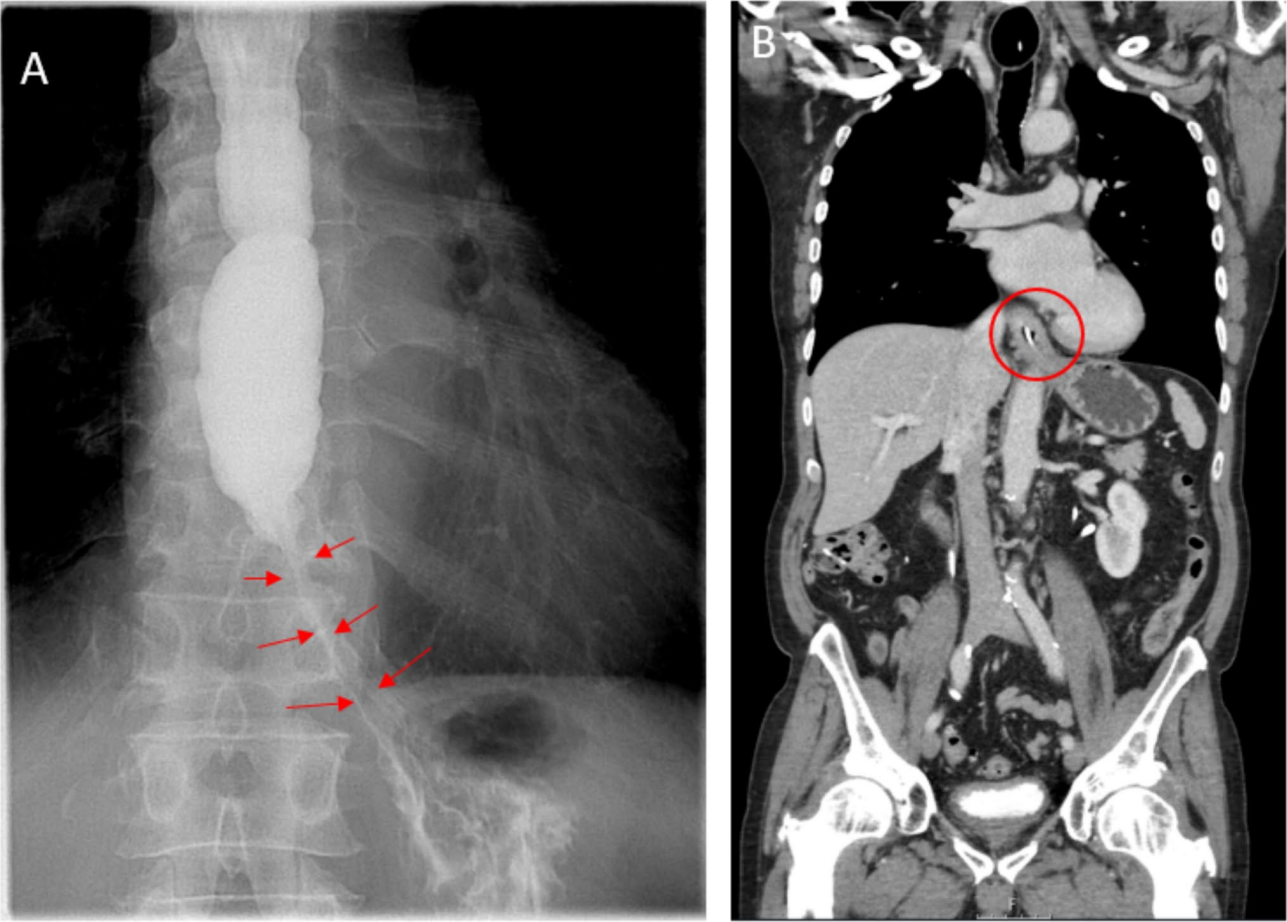
**A** The red arrows indicate a luminal narrowing in the distal esophagus on the barium swallow. **B** The area circled in red on the CT scan shows the distal esophagus with visualization of an inserted nasojejunal tube

Initial diagnostic workup included an upper endoscopy with stepwise biopsies of the esophagus. Macroscopic inspection during endoscopy was unremarkable, except for a whitish exudate in the distal esophagus and a possibly spastic appearance of the distal esophageal segment (see Fig. 1). There were no other features typically associated with eosinophilic esophagitis (EoE), such as rings, furrows, or stenosis. The histopathological examination (three specimen containers, including one with gastric biopsies, a second with biopsies from the distal esophagus containing three fragments measuring between 0.2 and 0.5 cm, and a third with one biopsy fragment from the proximal esophagus measuring 0.2 cm) was unremarkable, showing no evidence of eosinophilia in the mucosal epithelium. A barium swallow study revealed a markedly dilated esophagus (megaesophagus, see Fig. 2A), and a computed tomography (CT, see Fig. 2B) scan of the chest and abdomen showed no evidence of malignancy. Based on these findings, a primary diagnosis of achalasia was suspected, and the patient was referred for high-resolution esophageal manometry (HRM).

**Fig. 3:**
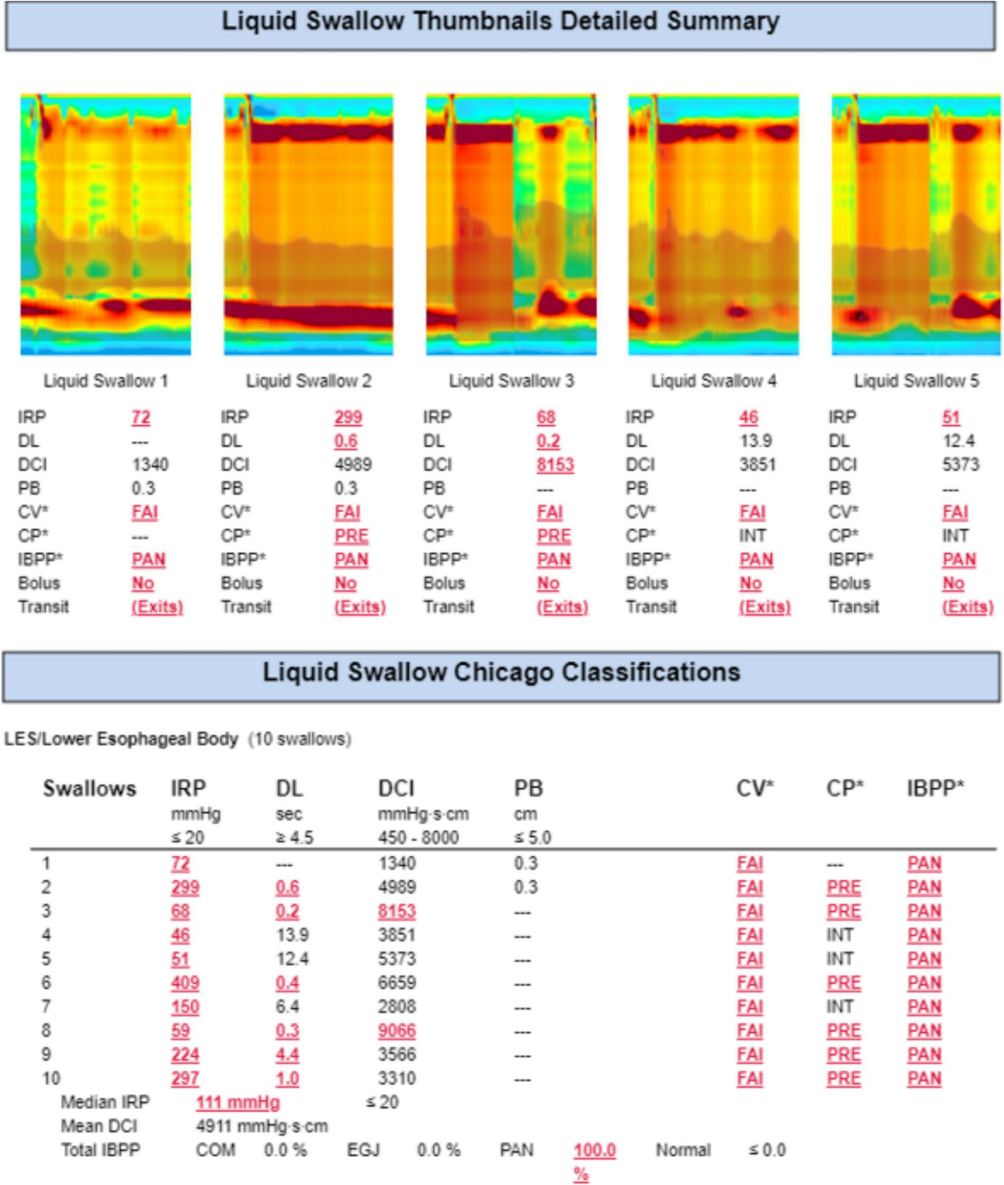
Excerpt from the high-resolution manometry with visualization of the swallows. Explanations: *IRP* integrated relaxation pressure, *DL* distal latency, *DCI* distal contractile integral, *pb* peristaltic break, *cv* contractile velocity, *cp* contractile pressure, *IBPP* intrabolus pressure pattern, *FAI* fail, *PRE* pre-contraction rise event, *PAN* pan-esophageal pressurization

HRM confirmed the diagnosis of achalasia type 2 [7]; however, it revealed an unusually elevated integrated relaxation pressure (IRP) and lower esophageal sphincter (LES) resting pressure of 244 mmHg (reference range < 15 mmHg) with a mean IRP of 111 mmHg, which are significantly higher than typical values for achalasia (see Fig. 3). With the pan-esophageal pressurizations also shown, this formally corresponds to achalasia type 2.

However, given these values, we suspected also pseudoachalasia due to a malignant process.

**Fig. 4:**
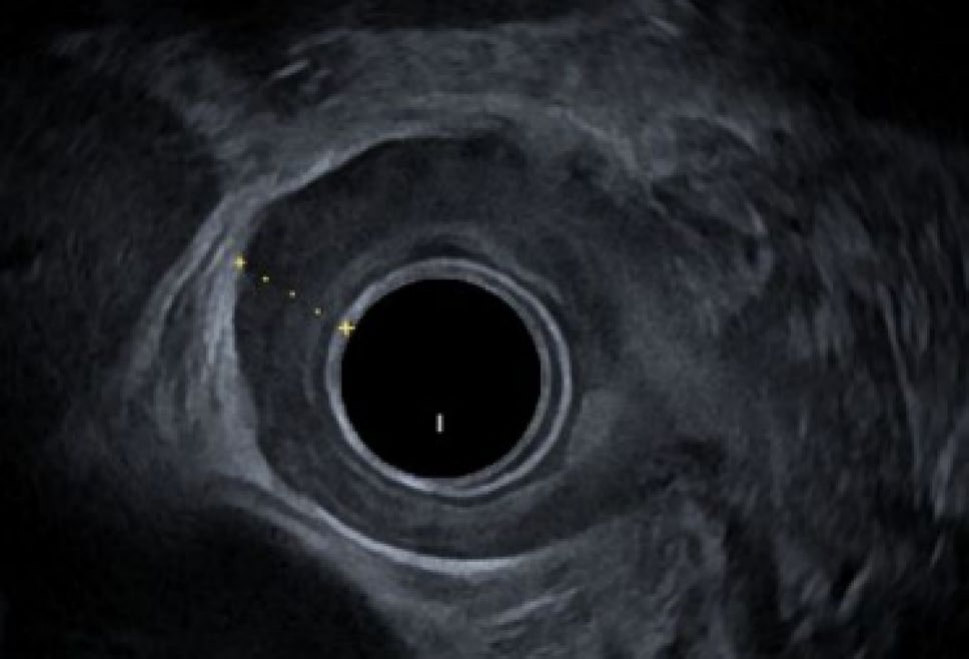
Upper endoscopic ultrasound (EUS) with visualization of the esophageal wall in the lower esophagus

A repeat CT of the chest and abdomen and an endoscopic ultrasound (EUS) were performed. Endoscopic ultrasound (EUS, see Fig. 4) demonstrated a thickened esophageal wall, measuring up to 8 mm in the distal esophagus, but did not reveal any mass or malignant lesion.

Although repeated EUS and CT imaging continued to show no evidence of malignancy, we could not definitively exclude a malignant process or an alternative diagnosis mimicking type II achalasia. Therefore, we proceeded with a Heller myotomy based on the following considerations: firstly, for diagnostic purposes, as the procedure would provide tissue for histopathological examination; and secondly, to offer definitive therapy in case the histology revealed no abnormalities. We opted against peroral endoscopic myotomy (POEM), as the patient was already on proton pump inhibitor therapy, and POEM in the context of preexisting or anticipated reflux is considered a relative contraindication.

**Fig. 5:**
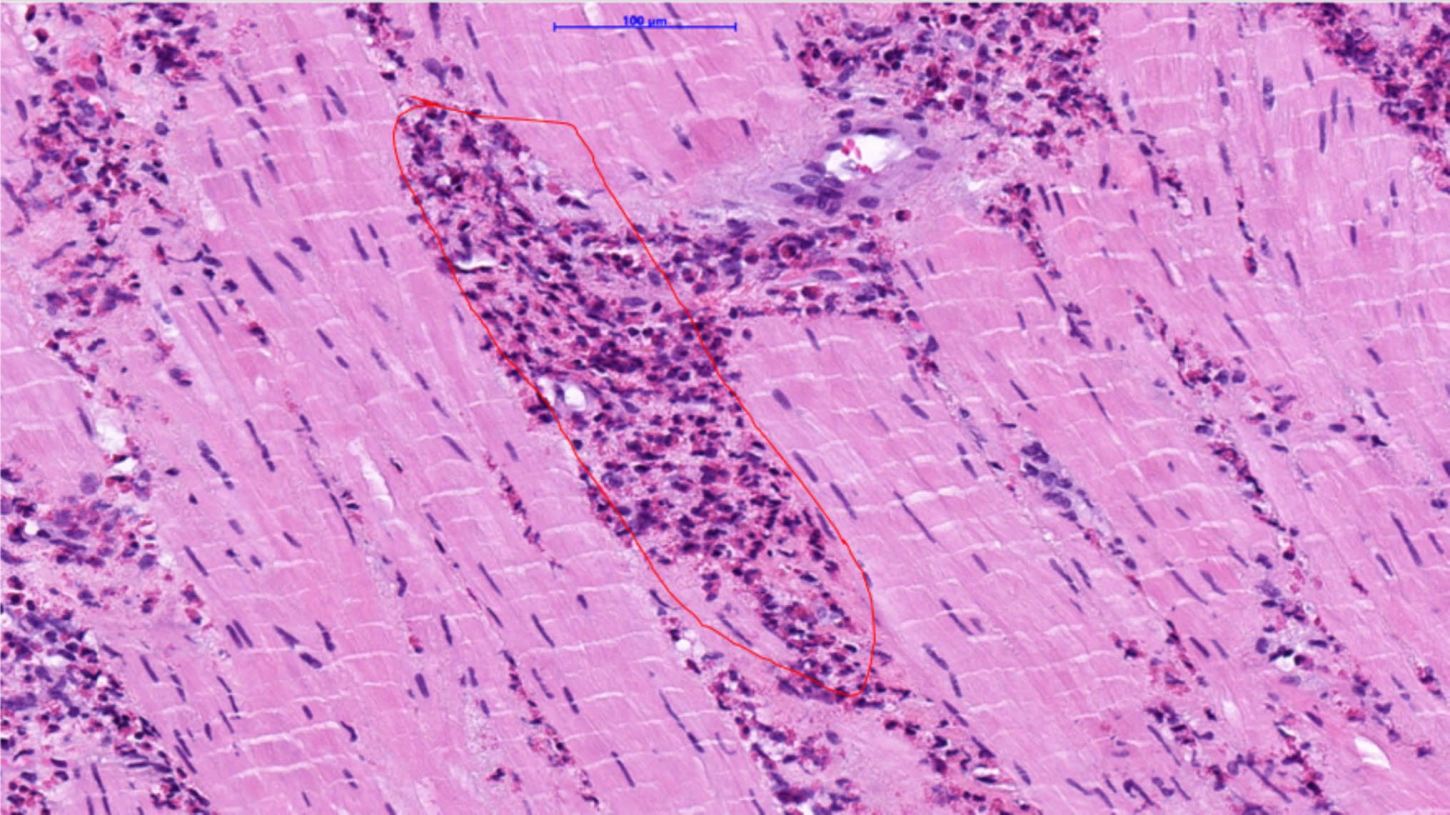
Histopathological specimen from the muscle biopsy obtained during Heller myotomy. The area outlined in red indicates the eosinophilic infiltration

Histopathologic analysis of the esophageal muscular layer from the Heller myotomy revealed a marked infiltration of eosinophils (> 100 per high-power field, see Fig. 5), confirming the diagnosis of eosinophilic esophageal myositis. The surgical specimen obtained during the Heller myotomy did not include mucosal or submucosal tissue, thus precluding further assessment of eosinophilic involvement in additional layers of the esophageal wall.

Based on these findings, high-dose corticosteroid therapy with prednisolone 40 mg daily was initiated on the 5th postoperative day.

On the 6th postoperative day, the 2nd day of steroid treatment, a barium swallow showed good passage.

**Fig. 6:**
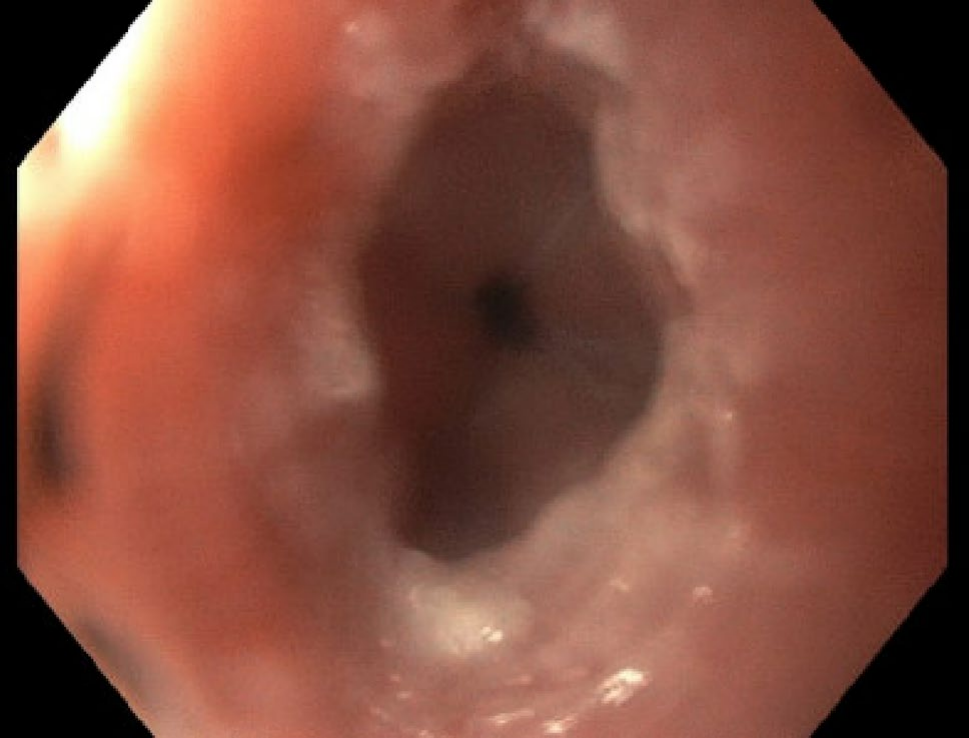
Follow-up endoscopy. Representation of the distal esophagus

Despite this initial improvement, the patient continued to experience dysphagia and required nasojejunal tube feeding for several days postoperatively. Systemic steroid therapy was continued for approximately 5–6 weeks and then gradually tapered until complete discontinuation. Additionally, he developed a urinary tract infection during steroid therapy, which was treated empirically with ciprofloxacin. Following the initiation of steroid therapy, the dysphagic symptoms rapidly regressed. In a primary care follow-up (5 weeks after the surgery and while still on prednisolone 40 mg once daily), a differential blood count was performed, which showed no abnormalities. After tapering off systemic corticosteroids, local therapy with budesonide at a dose of 1.5 mg per day was initiated. Symptoms with dysphagia and mucus reappeared about 1 week after the steroids had been fully tapered and under treatment with budesonide. In a follow-up gastroscopy 16 weeks after the Heller myotomy and on treatment with budesonide, a macroscopic suspicion of eosinophilic esophagitis was raised based on an EREFS score of 6 points (see Fig. 6). However, this was not confirmed by stepwise esophageal biopsies (four biopsy specimens from the distal esophagus, measuring up to 0.3 cm in size; four biopsy specimens from the proximal esophagus, measuring up to 0.5 cm in size), which did not show any mucosal eosinophils.

**Fig. 7:**
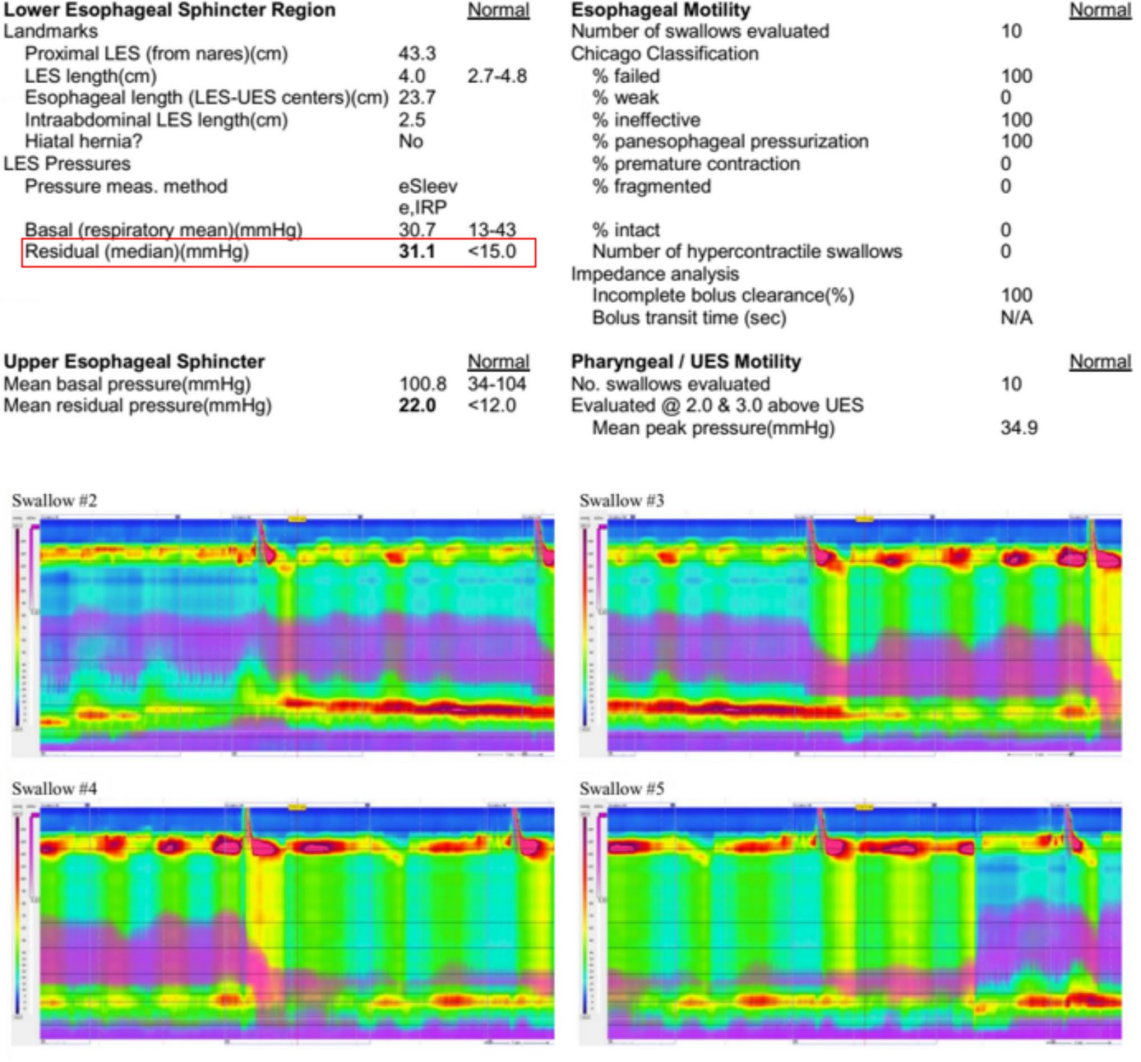
Repeat high-resolution manometry in the follow-up. Explanation: The median IRP is highlighted in red

A repeat manometry (also performed 16 weeks postoperatively and on treatment with budenosid) continued to show a pattern consistent with achalasia (see Fig. 7) [7]. Compared to the initial preoperative findings and before the initiation of systemic steroid therapy, there was a significant reduction in IRP and the amplitude of pan-esophageal contractions. Based on the findings described above (manometry and clinical symptoms), we initiated another escalation of therapy with prednisolone 40 mg once daily and discontinued the local budesonide treatment. Throughout the entire follow-up period, the patient received no medications other than oral steroids, intermittent budesonide, symptomatic treatment for nausea, and enteral nutrition with Fresubin via the nasojejunal tube—specifically, no empirical treatment of EoEM was administered.

## Discussion

Eosinophilic esophageal myositis (EoEM) is a rare entity characterized by eosinophilic infiltration confined to the muscularis propria of the esophagus, without involvement of the mucosal layer [2, 3], which distinguishes it histopathologically from eosinophilic esophagitis (EoE) [1]. Although EoE is well defined by mucosal eosinophilia (> 15 eosinophils per high-power field [HPF]) and characteristic endoscopic findings [1], EoEM often escapes detection by standard endoscopic biopsy because it affects deeper tissue layers exclusively. This distinctive pathology requires advanced diagnostic procedures, such as endoscopic ultrasound-guided fine-needle aspiration (EUS-FNA) or surgical muscle biopsies obtained during procedures such as peroral endoscopic myotomy (POEM) or laparoscopic Heller myotomy, as seen in our case [8, 9].

In addition to their distinct histopathological features, EoEM and EoE differ markedly in clinical and endoscopic presentation. EoE typically presents in younger patients with an atopic background and symptoms such as dysphagia, food impaction, and sometimes chest pain, accompanied by endoscopic signs like rings, linear furrows, and white exudates. In contrast, patients with EoEM are usually middle-aged men who present with pronounced esophageal motility disturbances, such as severe dysphagia or chest pain, often without any prior allergic history. Endoscopically, EoEM shows a normal mucosal surface or non-specific narrowing, without the typical features seen in EoE. These differences are clinically important, as the normal endoscopic and mucosal biopsy findings in EoEM may delay diagnosis unless deeper layers are sampled.

A comparison of these entities is summarized in Table 1.

**Table 1:** Comparision of achalasia, EoE and EoEM

Feature	Achalasia	EoE	EoEM
Age	Middle-aged to elderly adults	Typically younger male patients	Elderly, middle-aged adults
Comorbidities	Often non-atopic, possible neurodegenerative or idiopathic causes	Frequently present with atopic history	Typically absent
Symptoms	Progressive dysphagia to solids and liquids, regurgitation, weight loss	Dysphagia, food impaction, chest discomfort	Severe dysphagia, chest pain
Endoscopic findings	Often normal or showing esophageal dilatation	Rings, furrows, exudates	Normal mucosa or diffuse narrowing
Mucosal eosinophilia	Absent	Present (> 15 eos/HPF)	Absent
Muscularis propria involvement	Absent	Rare or absent	Prominent eosinophilic infiltration
Diagnostic tools	High-resolution manometry, barium swallow, endoscopy	Mucosal biopsies	EUS, full-thickness or myotomy-based biopsy
Therapy	POEM, Heller myotomy, pneumatic dilatation	Dietary therapy, topical corticosteroids, PPI, sometimes biologics	POEM and corticosteroids in case reports

This comparative summary highlights the need for clinical suspicion of EoEM in patients with esophageal symptoms, normal mucosal biopsies, and motility abnormalities, prompting consideration of deeper diagnostic evaluation.

In our patient, profound eosinophilic infiltration of the muscularis propria was unexpectedly identified after surgical myotomy performed to address severe dysphagia initially attributed to achalasia. The unusually high LES pressures observed on high-resolution manometry raised suspicion of a secondary obstructive pathology of malignant origin, later explained by eosinophilic myositis. This highlights the diagnostic complexity of EoEM, which clinically mimics primary esophageal motility disorders such as achalasia or jackhammer esophagus [2, 10].

The differentiation of EoEM from EoE and eosinophilic gastroenteritis (EGE) remains a subject of ongoing debate. EoE typically manifests with mucosal eosinophilia driven by allergen-specific Th2 immune responses, featuring endoscopic signs such as rings, furrows, and exudates [1]. In contrast, EoEM lacks these mucosal changes, and eosinophilic infiltration in mucosal biopsies is consistently negative [6]. Some authors like Sato et al. propose that EoEM could represent an atypical or advanced manifestation of EoE involving deeper tissue layers; yet, evidence for progression from mucosal to transmural involvement is lacking [6]. Conversely, EGE generally involves eosinophilic infiltration in multiple layers of the gastrointestinal tract beyond the esophagus, with systemic symptoms often including abdominal pain, vomiting, diarrhea, or peripheral eosinophilia, none of which were prominent in our patient or in other reported EoEM cases [2, 5, 9].

To date, approximately view adult cases of EoEM have been documented in the literature, underscoring its rarity while also providing insight into its consistent clinical features. Affected individuals are predominantly middle-aged men, presenting universally with dysphagia and commonly with chest pain [5, 10]. Endoscopic findings in these cases uniformly describe either normal mucosal appearance or diffuse esophageal narrowing without mucosal lesions. Advanced imaging, particularly EUS, reveals characteristic thickening of the muscular layer, and manometric findings often show hypercontractile or obstructive motility patterns [8].

Histologically, the hallmark of EoEM—intense eosinophilic infiltration limited to the muscularis propria—clearly differentiates it from EoE and EGE. Sato et al. [8] proposed specific diagnostic criteria for EoEM, emphasizing muscular eosinophilic infiltration with absence of significant mucosal eosinophilia.

Based on the HRM findings, achalasia must also be considered as an important differential diagnosis. In this case, the key distinguishing feature was the markedly elevated pan-esophageal pressure, which was significantly higher than typically observed in classic achalasia. The main differences between achalasia and EoEM lie in their underlying etiology—neurogenic versus eosinophilic inflammation—and treatment approach, as detailed in Table 1.

Treatment strategies for EoEM remain empiric due to limited cases and absence of established guidelines. Systemic corticosteroids are the most commonly used treatment and have consistently resulted in rapid symptomatic improvement [5, 9, 10]. In our patient, high-dose prednisone therapy initially restored esophageal passage and relieved dysphagia; however, the subsequent tapering led to relapse, indicating a potentially chronic or relapsing inflammatory process. This experience aligns with several other reported cases in which initial response to steroids was favorable, but prolonged therapy or maintenance regimens were necessary to sustain remission [3, 6]. Given the limitations and potential adverse effects of long-term corticosteroid therapy, dupilumab, a monoclonal antibody targeting the IL-4 receptor alpha, emerges as a promising therapeutic option. Dupilumab has demonstrated significant efficacy in eosinophilic esophagitis [11, 12] by reducing eosinophilic inflammation through inhibition of IL-4 and IL-13 signaling pathways [13], and thus could theoretically exert similar beneficial effects in EoEM. While no dedicated trials or case reports yet exist for EoEM specifically, the shared type 2 inflammatory mechanisms suggest that dupilumab may effectively suppress eosinophilic infiltration of the esophageal muscular layer [14]. This targeted biologic therapy might serve as a steroid-sparing alternative, especially in steroid-refractory cases or for patients at risk of steroid-related complications. Future studies are essential to evaluate the precise role and long-term efficacy of dupilumab in managing eosinophilic esophageal myositis.

Surgical or endoscopic interventions, such as POEM or myotomy, also play a dual therapeutic and diagnostic role in managing EoEM-associated motility disorders [2, 15]. In several reported cases, including ours, these interventions not only facilitated diagnosis through deep biopsy but also mechanically alleviated dysphagia by disrupting hypercontractile muscular segments [2, 10, 15]. Nevertheless, given the inflammatory nature of the disease, medical therapy with corticosteroids remains essential for sustained symptom control.

Patient perspectives from documented EoEM cases, including our patient, highlight the clinical burden associated with delayed diagnosis, prolonged dysphagia, weight loss, and therapeutic uncertainties. The initial diagnostic ambiguity and recurrent symptoms despite intervention significantly affect quality of life, emphasizing the need for increased clinical awareness and early consideration of deeper and full-thickness biopsy techniques in atypical esophageal motility disorders.

In conclusion, EoEM should be acknowledged as a distinct eosinophilic disorder of the esophagus, clearly differentiated histologically and clinically from both EoE and EGE. Although its exact pathogenesis remains unclear, the clinical management of EoEM requires a high index of suspicion, prompt histological confirmation through deep muscular biopsy, and an empirical treatment approach using corticosteroids. Further research, particularly prospective studies, is essential to establish clearer diagnostic criteria, clarify pathogenetic mechanisms, and determine optimal therapeutic strategies for this rare but clinically significant condition.
